# Drug delivery system for controlled release of empagliflozin from alginate-chitosan nanocarrier system

**DOI:** 10.55730/1300-0527.3370

**Published:** 2022-02-03

**Authors:** Nasim RAMAZANZADEH, Nosrat O. MAHMOODI

**Affiliations:** Department of Organic Chemistry, Faculty of Science, University of Guilan, Rasht, Iran

**Keywords:** Calcium alginate, chitosan, drug delivery, empagliflozin, nanocarrier

## Abstract

A biocompatible nanocarrier system was prepared in this research through the reaction of calcium alginate (CA) with chitosan (CS). The structure of developed nanocarriers (CS-CA) was characterized by thermogravimetric analysis (TGA), Fourier transforms infrared (FT-IR) spectroscopy, field emission scanning electron microscopy (FE-SEM), X-ray diffraction (XRD) atomic force microscopy (AFM), and transmission electron microscopy (TEM). Swelling properties of CS-CA and CA, and their ability for loading and in vitro release of empagliflozin (EMP) were also investigated. The results showed the higher loading capacity of CS-CA compared to CA. For both nanocarriers, the drug release was higher at neutral pH (7.4 and 6.8) when compared to acidic pH (1.2). Despite the higher release of CA than CS-CA, the latter exhibited a favorable sustained drug release in all pH levels. As a result, CS-CA nanocarrier (EMP@CS-CANC) can be suggested as a new candidate for colon drug delivery of EMP.

## 1. Introduction

Improvement of drug solubility, permeability, and bioavailability has remained one of the major challenges in their commercialization. In this regard, drug delivery systems have been developed as a promising approach [1,2]. Following the progression of nanotechnology, a new class of nanoparticles has been developed with diverse benefits such as improved drug solubility, reduction of required dose, sustained drug release, targeted delivery of drugs, and bioavailability [3,4]. Synthetic [5] and natural polymers [6,7], as well as their combinations [8], have been employed for drug delivery. Natural polymers such as gums, mucilages, and polysaccharides are nontoxic, biocompatible, inexpensive, and widely available. Among the polysaccharides, sodium alginate (SA) and chitosan (CS) have been extensively utilized for the delivery of different drugs such as a novel drug delivery system [9–14]. SA is a biodegradable and biocompatible natural polymer that can lead to coagulation in various drugs. SA is composed of (1–4)-linked-D-mannuronic acid (M) and -L-guluronic acid (G) in various arrangements and ratios. This biopolymer can form a hydrogel in the presence of divalent cations (e.g., Ca^2+^, Ba^2+^, Sr^2+^, and Zn^2+^). Such hydrogel structures can encapsulate the drug, allowing for designing DDS (drug delivery system) [15,16]. Several studies have focused on the development of calcium alginate (CA) beads for controlled drug delivery of orally administered drugs [17–19]. The CS is a linear, biological, and nontoxic polysaccharide in which D-glucosamine and *N*-acetyl-D-glucosamine units are connected by β-(1–4) glycosidic linkages. CS can be isolated by partial destruction of chitin. This natural polysaccharide has been extensively applied in DDS [20–22]. Crosslinking of CA and CS in a bead could be useful for medical and pharmaceutical investigation. Such hybrid systems can offer higher stability compared to their constituent polymers [23]. The application of CA and CS nanocarrier (CA-CS NC) in DDS has recently drawn great attention. For example, Nalini et al. synthesized SA/CS nanoparticles (NPs) for drug delivery which resulted in improved therapeutic effects and efficacy [24].

Empagliflozin (EMP; (4-chloro-3-(4-[(3S)-tetrahydrofuran-3-yloxy]benzyl))phenyl) is an orally administered antidiabetic drug capable of reducing the risk of heart failure or the progression of chronic kidney disease in patients with type-II diabetes. It can be prescribed instead of metformin and has shown several superiorities over sulfonylureas. It may be also used together with other medications such as metformin or insulin. Although not recommended for type-I diabetes, EMP may reduce the risk of death from cardiovascular disease in patients with type-II diabetes with cardiovascular comorbidity. Regarding recent trial findings, it is expected to be soon approved for patients with heart failure, irrespective of their diabetic status [25]. Therefore, in contribution to our previous studies [26–29], the main aim of this work is the loading of this drug on a nanocarrier based on the combination of CS and CA to improve the medicinal efficacy of EMP.

## 2. Experimental

### 2.1. Materials

CS, SA, and AcOH were supplied from Sigma Aldrich Company. CaCl_2_ anhydrate, phosphate-buffered saline (PBS), HCl, NaOH, and EMP were also purchased from Merck Company.

#### 2.1.1. Preparation of CA beads

The CA beads were prepared by gelation method, using Ca^+2^ as a crosslinking agent. A total of 0.1 g SA powder was first dissolved in 20 mL purified H_2_O and stirred at room temperature (RT) for 6 h. This solution was kept in a refrigerator for 24 h. It was then was dropwise added to 40 mL 1.25% CaCl_2_ under mild stirring, at RT. After 1 h, the beads were formed, which were washed with deionized-H_2_O and dried at 50 °C overnight [30].

#### 2.1.2. Preparation of Chitosan-calcium alginate (CS–CA) beads

A total of 0.1% SA solution was made by dissolving SA powder in H_2_O under magnetic stirring at RT. The solution of CS and CaCl_2_ was ready by dissolving 0.04 g CS and 0.5 g CaCl_2_ in 40 mL of 1 % AcOH. Then, the SA solution was dropwise added to CS and CaCl_2_ solution within 60 min and stirred at RT for 24 h. The obtained CS-CA beads were filtered, rinsed with distilled H_2_O several times, and dried at 50 °C overnight in an oven.

#### 2.1.3. Preparation of EMP-loaded CA-CS beads

**Figure 1:**
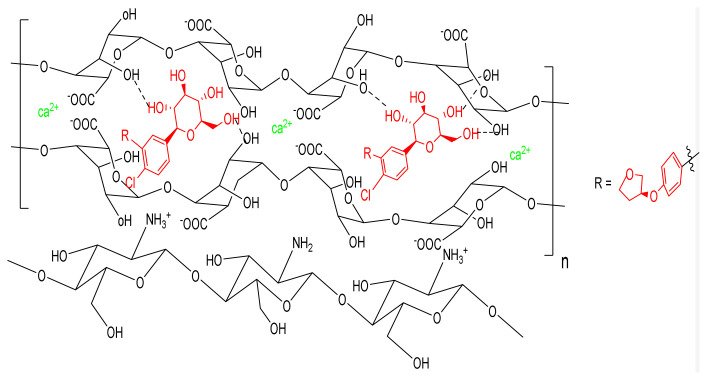
Schematic representation of preparation of EMP-Loaded CA-CS beads.

For loading of EMP on premade nanocomposite, 0.1 g SA powder was dissolved into 20 mL of EMP solution (250 ppm) and stirred at RT for 3 h. The solution pH was adjusted to 7.0 using 0.1 M NaOH. Then, 0.04 g CS and 0.5 g CaCl_2_ were dissolved in 40 mL of 2% AcOH and stirred for 24 h after adjusting the pH to 5.5 using 0.1 M HCl. Afterward, the solution of SA and EMP were dropwise added to CS and CaCl_2_ solution within 80 min, the reaction mixture was continuously stirred for 3 h at RT. Nanocarriers were precipitated at the bottom of the reaction vassal. Nanocarriers were washed with H_2_O to remove unreacted chemicals followed by drying in an oven at 50 °C overnight (Figure 1).

### 2.2. Characterization

Details on the characterization methods, swelling studies [31], and EMP loading efficiency [32] can be found in supplementary data.

#### 2.2.1. EMP release studies

To study the release behavior of EMP from nanocarriers, the release process was investigated at 37 °C via a dialysis bag with 5000 Da molecular cut off at three different pH. The temperature was kept at 37 °C using a water bath checked by the thermometer. The pH of the buffer solution was adjusted at desired values using a pH meter by the addition of diluted solutions of HCl and NaOH. The loaded nanocarrier (80 mg) was suspended in a phosphate buffer saline solution (PBS) (5 mL, pH 1.2, 6.8 and 7.4). Then it was poured into the dialysis bag and was incubated in PBS buffer (50 mL) of the same pH value at 37 °C under shaking (200 rpm/min). A certain value (3 mL) of solution from the container was removed after every time step, making sure to replace it with the same amount of fresh PBS solution. The amount of the released EMP was measured at different time points from 0 h to 12 h by a UV-Vis spectrophotometer (375.5 nm) [33]. % Release = [Release EMP/Total amount of EMP] × 100 (Eq. 3)

## 3. Results and discussion

### 3.1. Characterization of the nanocarrier

#### 3.1.1. FT-IR spectra

**Figure 2:**
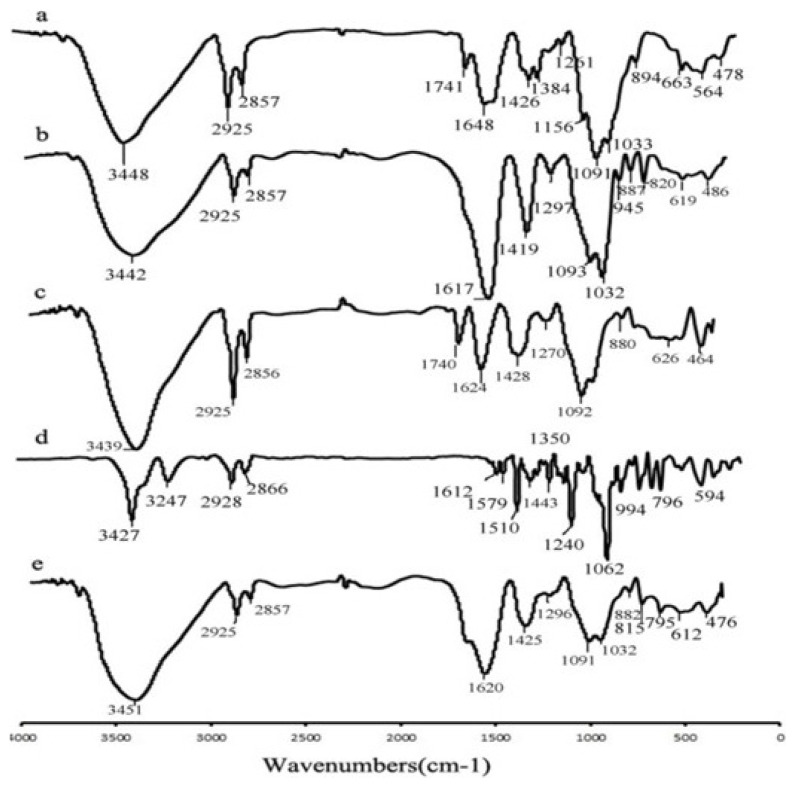
Profiles of FT-IR spectrum CS (a), SA (b), CS-CA (c), EMP (d), CS-CA-EMP (e).

Figure 2 represents the FT-IR spectra of CS, SA, CS-CA, EMP, and EMP@CS-CANC. CS spectrum showed a broad peak at 3400 cm^−1^ which can be assigned to N–H and O–H stretching vibrations in the CS molecules. The peak at 2800–2950 cm^−1^ can be attributed to the C–H stretching vibrations. The peaks at 1648 and 1741 cm^−1^ also correspond to NH and C=O amide bending vibrations, respectively. The peaks at 1156, 1091, and 1033 cm^−1^ can be ascribed to asymmetric vibrations of C-O-C. In the FT-IR spectrum of SA, the broad peaks at 3400, 1416, 1617, and 1297 cm^−1^ can be assigned to O–H stretching, asymmetric symmetric and symmetric stretching of the –COO, and stretching of C-O groups [34]. Concerning the FT-IR spectrum of nanocarrier CS-CA, characteristic symmetric stretching peaks of –COO shifted from 1617 to 1624 cm^−1^, suggesting the formation of an ionic bond between calcium ions and carboxylic ions, the peaks were slightly broadened in the regions of 1400 and 900–1100 cm^−1^. These changes showed the interactions between SA, Ca^+2^, and CS. The spectrum of EMP indicated the bands at 3400, 3000, and 2800–2900 cm^−1^ which are related to the OH, =CH of the aromatic ring and –CH_2_ groups, respectively. Regarding the presence of aromatic rings and ether groups in the structure of EMP, the corresponding peaks appeared in the regions between 1400–1600 and 1000–1200 cm^−1^.

Finally, the spectrum of the drug-loaded sample (EMP@CS-CANC) showed all the expected vibration bands of its constituents, confirming their contribution in the final nanocarrier. In this spectrum, the peaks in the region of 3000–3400, 1600–1700, 1400–1500, and 1000–1100 cm^−1^ were broadened probably due to the electrostatic interactions and the formation of hydrogen bonds between functional groups on the nanocarrier and EPA molecule.

#### 3.1.2. XRD patterns

The crystallographic structure of the nanocarrier was determined by XRD. Figure S1 shows the XRD patterns of CS, SA, and CS-CA. The typical peak (a) of CS can be observed at 2Θ = 20–22°, representing the amorphous structure of CS. The X-ray pattern of SA (b) exhibited two peaks at 2θ = 13° and 22°. Finally, curve (c) shows the XRD patterns of the nanocarrier. In addition to peaks of SA and CS at 2θ = 13–15° and 19–22°, one expanded peak emerged at 2θ = 40° that probably corresponds to the presence of calcium ions in the nanocarrier [35–37].

#### 3.1.3. Thermal study

Thermal analysis has been widely used to assess the thermal features of polymeric materials. The TGA results of CS, SA, and CS-CA can be found in Figure S2. A slight weight loss can be observed in the first stage (below 150 °C) of CS degradation (a) which can be attributed to evaporation of H_2_O. The second stage at 250–350 °C involved a significant weight loss due to the decomposition of the CS backbone. The primary weight loss of SA (b) occurred below 130 °C which can be ascribed to the evaporation of the adsorbed H_2_O. The maximum weight loss temperature was in the range of 220–250 °C, corresponding to the decomposition of the organic structure. Concerning the nanocarrier (c), the first weight loss below 150 °C can be attributed to the desorption of volatile compounds, mainly moisture. The second weight loss started at 200–330 °C with a milder slope compared to SA and CS which can be related to the decomposition of nanocarrier encompassing SA, CS, and calcium ions with electrostatic interactions and the formation of hydrogen bonds [22].

#### 3.1.4. FE-SEM and EDX analyses

The surface morphology of the CS-CA (Figure S3a) and EMP@CS-CANC (Figure S3b) was confirmed using FE-SEM (Figure S3). The different morphology of the EMP@CS-CANC (Figure S3b) as a regular interconnected spherical lattice structure compared to the regular and spherical structure of CS-CA (Figure S3a) is probably due to the drug loading on the nanocarrier and the accumulation of particles. After preparation of nanocarrier with the addition of calcium and CS solution to CA solution, as it can be seen from the image of nanocarrier (Figure S3b), it is obvious that some spherical lattices beads that tightly joined together and form aggregates. This morphology probably corresponds to the interaction between the content materials of the nanocarrier.

#### 3.1.5. TEM and AFM analysis

**Figure 3:**
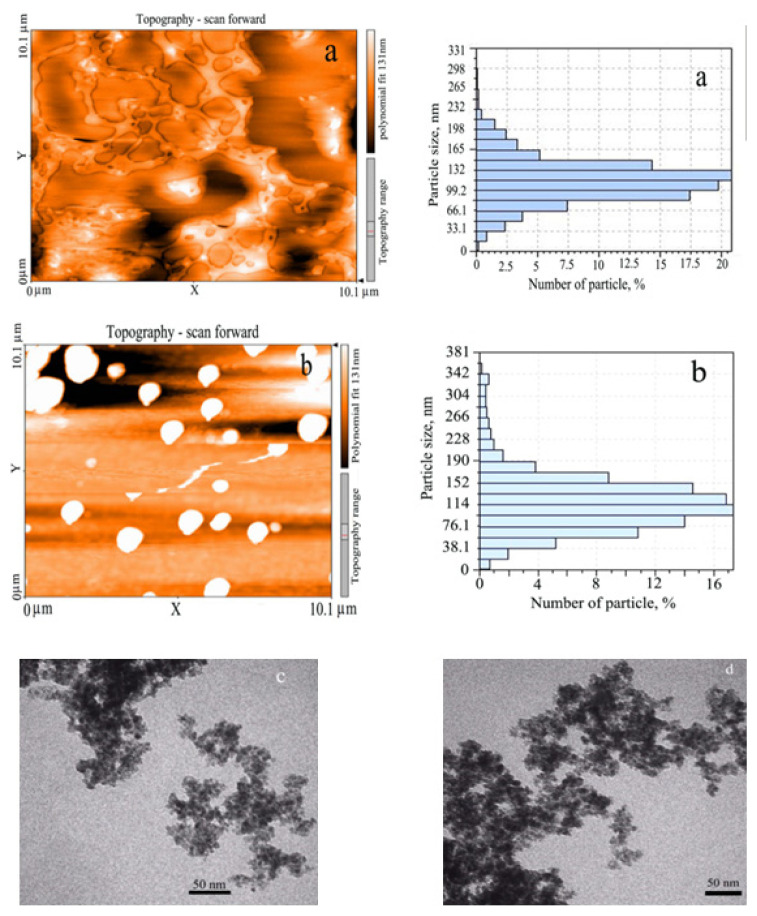
AFM (a,b (drug)) and TEM (c,d (drug)) image of CS-CA and CS-CANC-EMP.

The AFM and TEM images in Figures 3a–3d show that the average particle size is in the range of 66–132 nm for the nanocarrier and 76–152 nm for the nanocarrier containing the EMP-drug, respectively. Comparison of these images shows that the loaded drug has no significant effect on the morphology of the nanoparticles. However, the average particle size of nanocarrier increases slightly by loading drug on CS-CANC.

#### 3.1.6. Swelling studies

**Figure 4:**
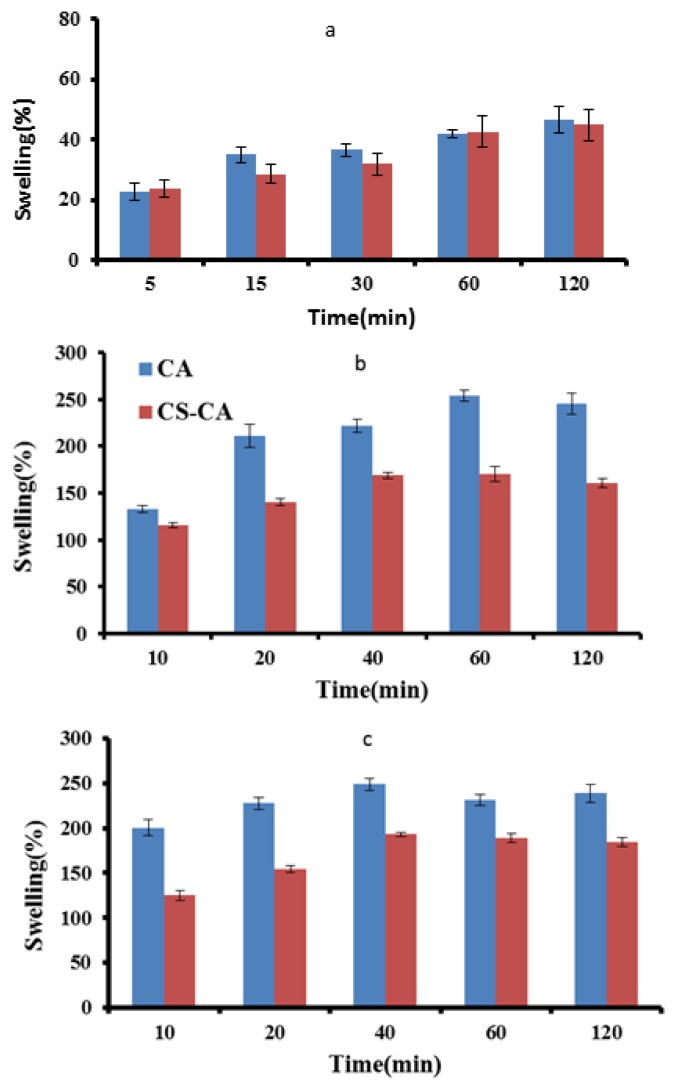
(a) Swelling characteristics of CS-CA and CA in a solution at pH 1.2 for 2 h and at 37 °C ±1, (b) swelling characteristics of CS-CA and CA in a solution at pH 6.8 for 2 h and at 37 °C ±1, (c) swelling characteristics CS-CA and CA in a solution at pH 7.4 for 2 h and at 37 °C ±1.

Some natural polymers such as SA, CA, and CA-CS polymers have promising swelling potential upon contact with fluids at various pH levels. This property can be assigned to the presence of hydrophilic groups on their structure. Upon fluid adsorption, the polymer chain can swell with no damage to the structure. This factor is very important in drug delivery; therefore, the swelling behavior of CA, CS-CA was investigated in PBS at three pH levels (1.2, 6.8, and 7.4) to simulate gastric and intestinal environments. Figure 4 shows the swelling behavior of the samples.

At pH=1.2 (Figure 4a), no alteration can be observed in the swelling ability of CA and CS-CA, the ester groups of SA were protonated to alginic acid. The insolubility of this compound under these conditions and its ability to form hydrogen bonding (H-bonding), inhibit the swelling of the nanocarrier beads. Similar swelling behavior was observed for CS-CA. Under acidic conditions, CS exhibited high solubility and its amine units converted into soluble NH^4+^ ions. However, the interaction of amino groups and ester groups was not sufficiently strong to initiate swelling. Thus, the restricted total swelling behavior is mainly governed by the CA structure. Figures 4b and 4c show the swelling ability of CA and CS-CA at two pH levels (6.8 and 7.4). As shown, prolonging the contact time from 5 to 60 min enhanced the swelling of the systems after which it remained approximately constant. Such a trend is attributable to ion exchange reactions between Na^+^ content of the PBS and Ca^2+^ coordinated to COO groups of SA. This ionic displacement altered the structure by creating a gap between the carbohydrate chain, promoting the penetration of liquid and swelling of the system. This process continues until the osmotic pressure of the beads equilibrates the strength of the crosslinking bonds and physical entanglements, electrostatic attractive forces, and H-bonding, leading to a new structure of the beads. As a result, the nanocarrier starts to decompose loses its mass. The swelling studies indicated that CS-CA beads are a more robust structure compared to CA, possibly due to the interactions among CS and CA chains. The maximum swelling degree of CS-CA systems could reach a swelling balance within about 40 min and maintain a constant weight till the end of the test. The interactions among the two polymers are responsible for the formation of nanocarrier particles with significant mechanical resistance, that limits the liquid uptake and the structure breakdown. These results are in line with other studies on different samples which reported an increase in swelling upon elevating the pH level [34–37].

#### 3.1.7. EMP loading efficiency and pH effects

The most important concern in the novel DDS is the drug loading on nanocarriers. In this regard, the pH is an essential factor affecting the efficient loading of the drug from aqueous solutions. The dependence of drug adsorption on the pH factor is closely connected with the type of surface functional groups on the carrier as well as drug solution chemistry. Therefore, in this work, EMP@CA and EMP@CS-CANC were studied at different pH values (3–9). The optimal pH was found to be 6.2 where the loading efficiency reached 58.48% and 81.65% for CA and CS-CA, respectively. This observation was related to the surface properties of nanocarrier at pH = 4 where CS is mainly protonated, while SA, as well as drug, is unionized. As a consequence, drug and surface weakly interact, leading to low drug loading. An increase in pH (above 4) gave rise to the deprotonation of -COOH on SA (pKa approximately 3.4–4.4) to -COO^−^. -COO^−^ could form complex with CS and establish a stable structure capable of interacting with the drug through H-bonding and electrostatic interaction, hence, incrementing the drug loading percentage. At higher pH levels, CS was deprotonated, leading to partial destruction of polyelectrolyte complexes and decreased EMP loading.

#### 3.1.8. In vitro release study

**Figure 5:**
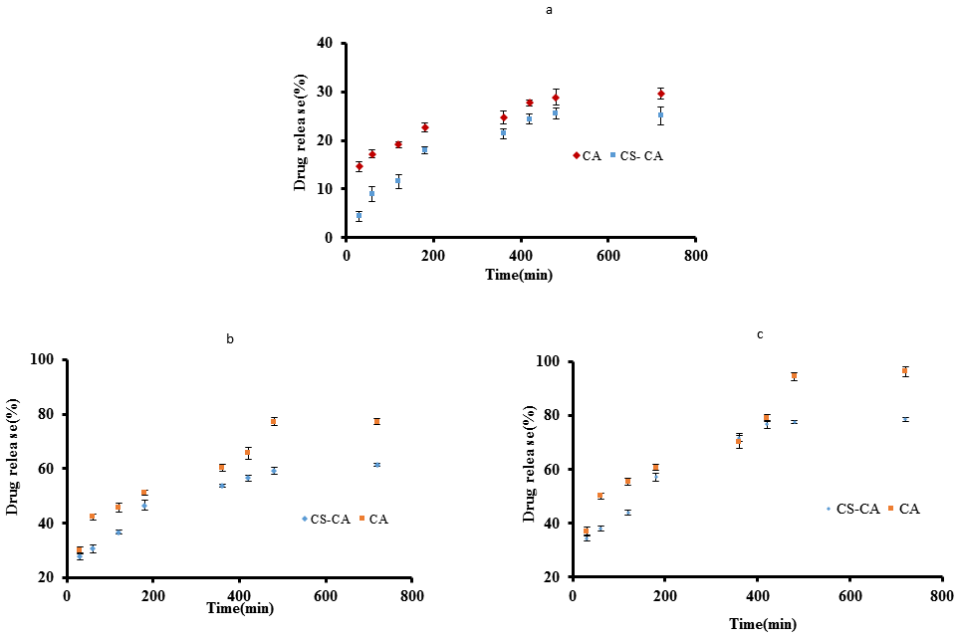
(a) Curves of EMP release from CA and CS-CA in an acidic environment with pH 1.2, (b) curves of EMP release from CA and CS-CA in PBS at pH 6.8, (c) curves of EMP and CS-CA in phosphate buffer at pH 7.4.

The release profile of EMP from CA and CS-CA is depicted in Figure 5 at a pH of 1.2, 6.8, and 7.4. In the acidic medium, the EMP released within 2 h at a quite low level (11.59%–19.16%) (Figure 5a).

The delay (postponement) in the release can be attributed to the reduced swelling ability of the nanocarrier. These results indicate that to minimize EMP delivery in an acidic condition it is better to promote and favor this procedure at the intestinal pH level [37].

Figure 5b shows the EMP release in PBS at pH 6.8. The systems primarily swelled and then started to erode and disintegrate, as a result, CA managed to release 77% EMP during approximately 8 h. During the same period, the amount of drug release from CS-CA particles was 59%. Such a difference can be attributed to the stronger electrostatic interaction and H-bonding between functional groups of the CA, CS, and EMP compared to pure CA.

Figure 5c illustrates the drug release from CA and CS-CA particles at pH 7.4. No significant difference was observed in the drug release from CA and CS-CA compare to pH of 6.8, although more quantities of the drug were released. Drug release rates were 94% and 77% for CA and CS-CA, respectively.

Such increases in the drug release are probably related to the partial destruction of polyelectrolyte complexes between CS and CA. At pH = 6.8, the drug release rate decremented. In PBS, CS-CA particles required longer times to deliver EMP as compared to CA. The presence of the CS-CA complex may cause a crumpled and irregular structure, hindering the drug release.

## 4. Conclusion

This study is the first attempt exploring the possibility of loading an antidiabetic drug (EMP) on CA and CS-CA nanocarriers using the ionotropic pregelation technique. The structure of the nanocarrier was assessed by FTIR, XRD, TGA, AFM, TEM, and SEM techniques. The maximum EMP loading on CS-CA (81.65%) was achieved at pH 6.2. The swelling evaluations at three different pH levels indicated that the highest swelling at pH = 7.4. The EMP release profiles suggested that although a part of EMP was released after contact of the carrier with gastric-simulated fluid, the EMP was broadly released at intestinal pH. The maximum EMP release from CA and CS-CA at pH = 7.4 was 94% and 77% after 8 h, respectively. These results indicated that the presence of CA in the structure of CS-CA nanocarriers led to the reverse release of the beads and increased capsulation efficiency. Therefore, CA and CS-CA can be suitable polymeric carriers for the controlled release of EMP.

## Supplementary data

### Characterization

The FTIR spectrophotometer (8400 S, Shimadzu) was applied to seek structural information in the limited area 4000–400 cm^−1^. The surface morphology was inspected by a scanning electron microscope (SEM) (Hitachi S4160 model) fitted with an energy dispersive X-ray analyzer (EDAX). TGA of nanocarriers was investigated using the LENSES STAPT-1000 calorimeter (Germany) by scanning up to 700 °C with a heating rate of 10 °C/min. X-ray diffraction (XRD, Shibuya-ku, Japan) was recorded at RT on a RigakuD/Max-2550 powder diffractometer with a scanning rate of 5°/min, in the *2θ* range of 10–70. AFM, model: Nano Wizard II NanoScience AFM, JPK Instruments Inc., Germany was applied to investigate morphology of nanocariers. TEM were performed using TEM microscope (Philips CM120).

### Swelling study

The swelling specifications of CA and CS-CA beads were defined by plunging dried test samples to swell in 5mL of a solution at pH 1.2 in 37 °C for 2 h and then added into a pH 6.8 and 7.4 medium, simulating gastrointestinal tract conditions. At particular time intervals, samples were removed from the swelling medium and were blotted with a piece of paper towel to absorb excess H_2_O on the surface. The swelling ratios (Qs) of test samples were calculated from the following expression [28]:


(Eq.1) Qs=(Ws-Wd)/Wd,

where Ws is the weight of the swollen test sample and Wd is the weight of the dried test sample. All experiments were conducted in triplicate.

### EMP loading efficiency

To calculate the EMP loading efficiency (LE), nanocarriers were filtered from the reaction mixture. The clear supernatant was analyzed for EMP content using PG Instruments T80UV-Visible spectrophotometer at 375.5 nm and the following equations [29].


(Eq. 2) LE (%)=[(Total amount of E​MP-the free amount of EMP in the supernatant)/Total amount of EMP]×100

Figure S1 X-ray diffractogram of CS (a), SA (b), and CS-CA (c).

Figure S2 TGA of CS (a), SA (b), and CS-CA (c).

Figure S3 FE-SEM analysis of CS-CA (a), CS-CA-EMPNC (b), and EDAX, CS-CA (c), CS-CANC-EMP (d).
