# Supplementary figures and images for: Drug delivery system for controlled release of empagliflozin from alginate-chitosan nanocarrier system
Source: Turk J Chem. 2022 Feb 3;46(3):805–13. doi: 10.55730/1300-0527.3370 (PMC10503968; doi:10.55730/1300-0527.3370)

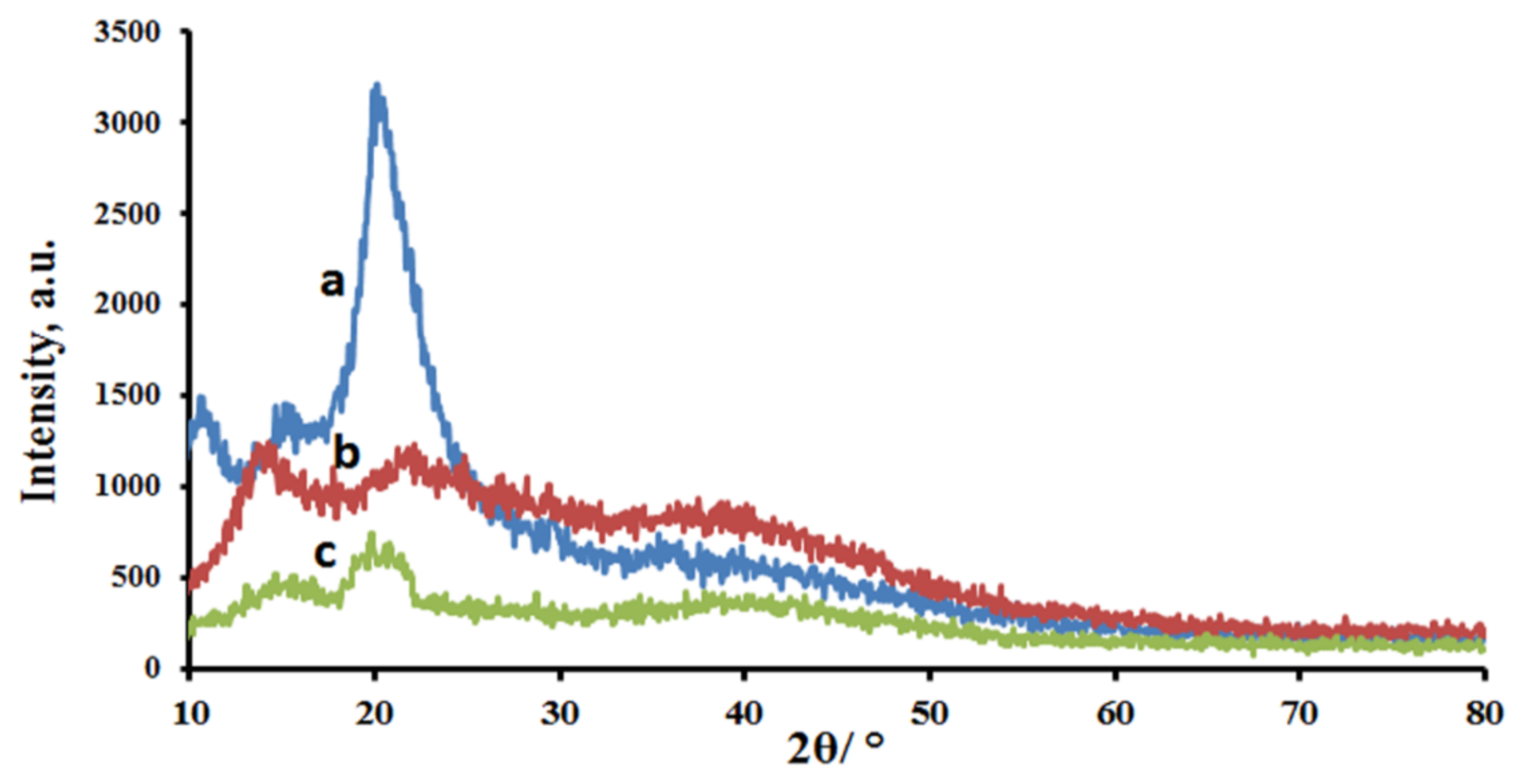

Supplement: Figure S1 — X-ray diffractogram of CS (a), SA (b), and CS-CA (c). [file turkjchem-46-3-805s1.tif]

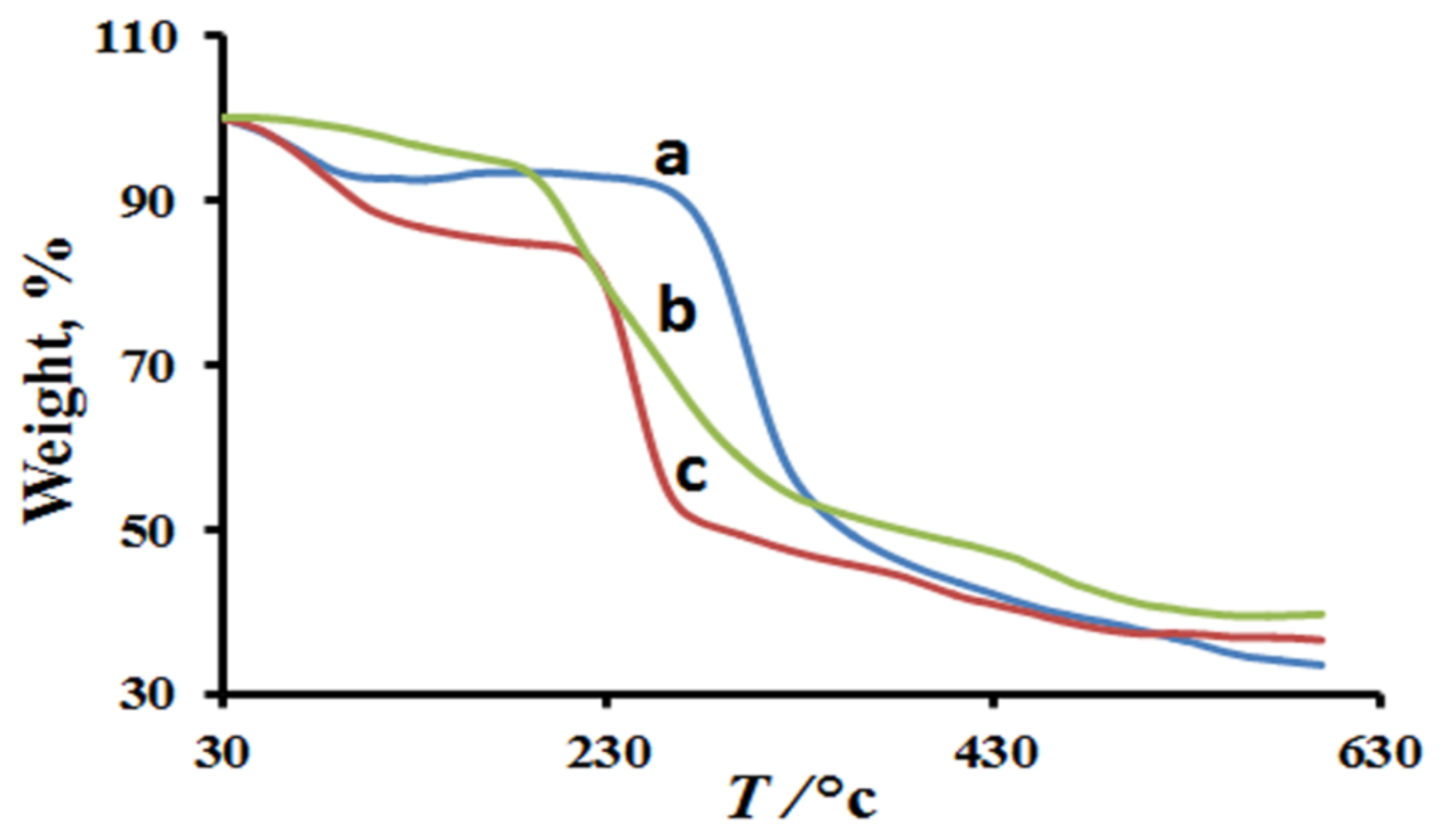

Supplement: Figure S2 — TGA of CS (a), SA (b), and CS-CA (c). [file turkjchem-46-3-805s2.tif]

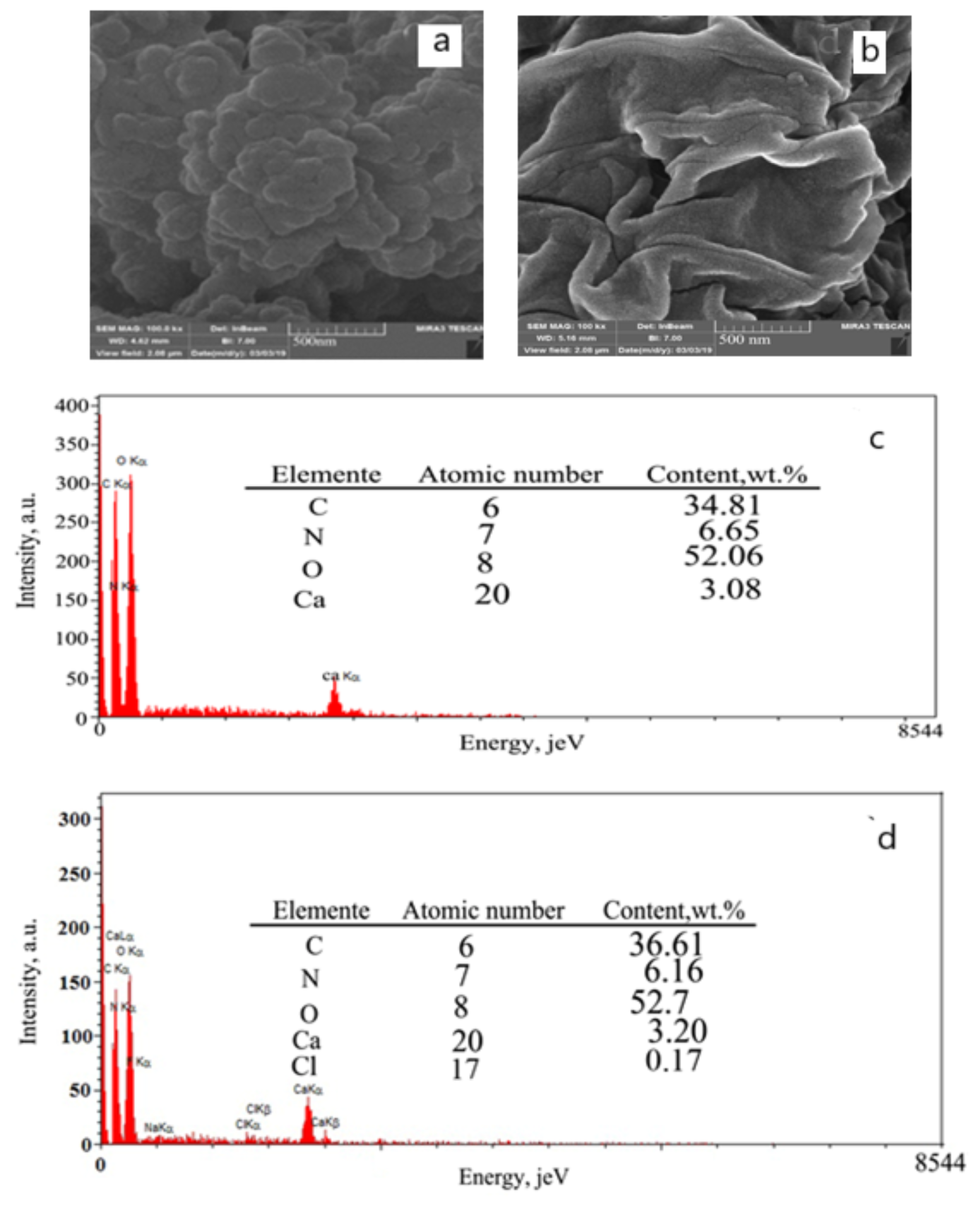

Supplement: Figure S3 — FE-SEM analysis of CS-CA (a), CS-CA-EMPNC (b), and EDAX, CS-CA (c), CS-CANC-EMP (d). [file turkjchem-46-3-805s3.tif]
